# Different Phenotypic, Photosynthetic, and Physiological Responses to Flooding between *Q. nuttallii* and *Q. palustris*

**DOI:** 10.3390/plants13121658

**Published:** 2024-06-15

**Authors:** Tiantian Sun, Mengzhu Wang, Xin Li, Yongxia Chen, Wangxiang Zhang

**Affiliations:** 1 College of Forestry, Nanjing Forestry University, Nanjing 210037, China; suntiantian1220@163.com (T.S.); wangmengzhu1214@163.com (M.W.); 2220100023@njfu.edu.cn (X.L.); 2 Co-Innovation Center for Sustainable Forestry in Southern China, Nanjing Forestry University, Nanjing 210037, China; 3 College of Civil Engineering, Nanjing Forestry University, Nanjing 210037, China

**Keywords:** flooding stress, *quercus*, physiological response, antioxidant defense, phytohormone

## Abstract

Flooding stress is an increasingly serious problem in wetlands, often affecting large areas of crops and timber production areas. The current study aimed to explore the species differences in responses to flooding stress between *Q. nuttallii* and *Q. palustris* in an outdoor environment. All the tested plants survived after a 60-day flooding treatment that left 5 cm of water above the soil surface. This suggests that the two species are flood-tolerant, so they can be applied in the construction of riparian protection forests and wetland restoration. Compared with control conditions, flooding treatment significantly decreased seedling height and diameter and the P_n_, G_s_, T_r_, F_v_/F_m_, ABS/CS_m_, TR_0_/CS_m_, ET_0_/CS_m_, RE_0_/CS_m_, IAA, and GA_3_ content and significantly increased the content of MDA, H_2_O_2_, soluble sugars, SOD, POD, ADH, ABA, and JA. Under control conditions, *Q. nuttallii* showed significantly greater growth and photosynthetic capability than *Q. palustris*. In contrast, *Q. palustris* exhibited less inhibition of growth and photosynthesis, oxidative stress levels, and antioxidant enzyme activities than *Q. nuttallii* under flooding conditions. The findings indicate that *Q. palustris* has better defense mechanisms against the damage caused by flooding stress than *Q. nuttallii*. *Q. nuttallii* was more sensitive and responsive to flooding than *Q. palustris*.

## 1. Introduction

Flood stress caused by climate change and human activities related to heavy rainfall, poor irrigation, and drainage practices is an increasingly serious problem in wetlands [1,2,3]. The land area affected by flooding is more than 17 million km^2^ worldwide [4]. Excessive soil water reduces the oxygen diffusion rate, as gases diffuse into water at a 10^4^ lower rate than in air [5]. After inundation with water, the soil environment becomes hypoxic as residual oxygen is consumed by the respiration of roots and microorganisms [5,6]. Flooding is becoming a significant environmental stressor affecting the production of crops and woody plants in large areas [7]. In the last 50 years, the area of arable land per capita has decreased from 0.32 ha to 0.21 ha due to climate change and the growing world population, and this area is expected to shrink further to 0.16 ha per capita by 2030 [5]. Therefore, understanding how plants, especially woody plants, function under flooding conditions is critical to introducing or improving flood-tolerant forage species in production.

Depending on the tolerance of plants, the greatest challenge posed by flooding is its ability to severely damage plant cells and organs and, ultimately, restrict physiological functions due to the lack of oxygen supply [8,9]. Anatomical changes and low O_2_ stress avoidance help plants tolerate floods [7,10,11]. Plants using the former strategy may try to avoid prolonged hypoxia by changing their morphological and anatomical adaptations, which may improve tissue regeneration and plant survival [4,12]. Plants using the latter strategy can conserve energy and carbohydrates to prolong survival by slowing down metabolic processes until a flooding event is over and the plant tissue enters a steady state [11,13]. Maintaining alcoholic fermentation under flooding conditions is crucial for anoxia tolerance in woody plants as it ensures the generation of ATP during flooding [10]. However, fermentation must proceed at a higher rate than respiration due to the higher amount of energy produced in mitochondrial respiration (38 mol of ATP per mol of glucose) compared with alcoholic fermentation (2 mol of ATP per mol of glucose) [10,12]. Consequently, flooded roots of plants need more carbohydrates, which could be essential for plant survival in prolonged hypoxic conditions.

Oaks (*Quercus* L.) are a major component of subtropical and temperate forests in the northern hemisphere. There are approximately three hundred species in this genus [14]. The oak is a dominant forest species in China, playing a key role in maintaining the biodiversity and stability of its ecosystems [14,15]. Previous studies reported that the cultivation of oaks is becoming increasingly important as a stabilizer of ecosystems with increasing climate extremes [16]. Nevertheless, the quality of oak seedlings is inconsistent, resulting in suboptimal reforestation of oaks [17]. The slow growth of the initial aboveground seedlings of oak species, high rates of acorn predation, and growth inhibition by flooding were cited as possible causes of oak regeneration problems in lowland deciduous forests [18,19]. Uncovering the potential physiological mechanisms involved in flooding is crucial for selecting and cultivating flood-tolerant plant species.

The sensitivity of oaks to flooding has been reported to vary widely. The white oak (*Quercus alba* L.) and water oak (*Quercus nigra* L.) showed similar physiological responses to flooding, whereas the duration of flooding required to induce physiological changes varied by species [16,20]. The marginally flood-tolerant sessile oak (*Quercus petraea* (Mattuschka) Liebl.) showed two distinct phases of flood response, during which physiological changes in seedlings predominated for the first three days, and substantial morphological and anatomical shifts also occurred during this period [16,21]. Previous studies reported that *Q. nuttallii* and *Q. palustris* showed resistance to waterlogging, which is desirable from both wildlife and timber production perspectives [22,23,24]. However, whether the two different oak species’ physiological response patterns and flood resistance are uniform remains unclear. Therefore, studies of different time points in physiological parameters including photosynthesis, biochemistry, and phytohormones in different species are needed to explore the causal relationship between the flooding tolerance and physiological parameters of these two oak species. Such studies will provide a knowledge base for selecting tree species that can adapt to climate change.

This study was conducted to determine the dynamic physiological responses of oak species (*Q. nuttallii* and *Q. palustris*) to flooding stress and the consistency of these responses. In addition, the causal relationship between flooding tolerance and the physio-biochemical parameters of oak genotypes was investigated. We predicted that (i) flooding stress would inhibit plant growth and leaf photosynthesis, decrease stomatal conductance and F_v_/F_m_, and increase MDA, H_2_O_2_, antioxidant enzyme, and ABA content. (ii) Since *Q. nuttallii* performs better under natural conditions than *Q. palustris*, we expected *Q. nuttallii* to perform better under flooding conditions.

## 2. Results

### 2.1. The Impact of Flooding on Plant Growth and Leaf Color

**Figure 1:**
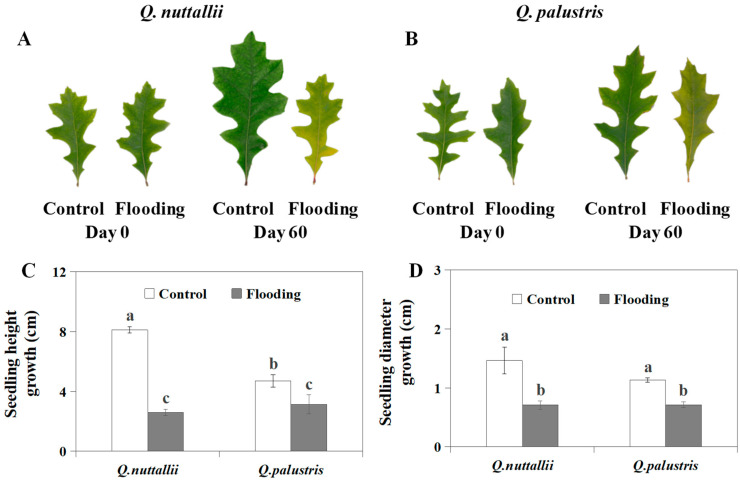
Leaf phenotype and seedling growth under flooding conditions for *Q. nuttallii* and *Q. palustris*. (**A**) Leaf phenotype of *Q. nuttallii*. (**B**) Leaf phenotype of *Q. palustris*. (**C**) Seedling height growth of *Q. nuttallii* and *Q. palustris*. (**D**) Diameter growth of *Q. nuttallii* and *Q. palustris*. The bars show the mean ± SE (n = 3). Mean values with different letters are statistically different at the 5% level.

*Q. nuttallii* had faster growth rates than *Q. palustris* under control conditions (Figure 1 and Table 1). Under flooding conditions, *Q. nuttallii* exhibited greater seedling growth inhibition than *Q. palustris*. Compared with the control, both species showed significantly inhibited seedling growth rates regarding height and diameter after being exposed to flooding stress for 60 days (Figure 1 and Table 1). These results suggest that *Q. nuttallii* exhibited greater growth under control conditions, while *Q. palustris* showed lower growth inhibition under flooding conditions.

**Figure 2:**
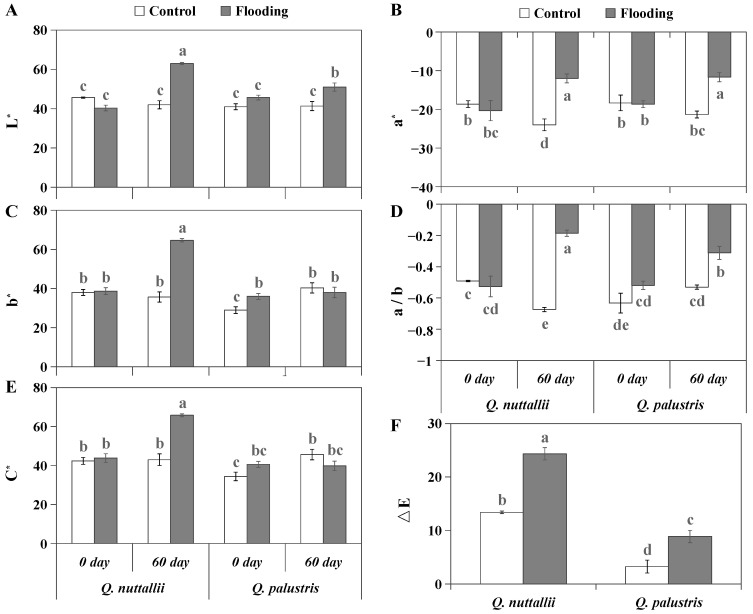
Leaf color parameters in CIELAB space for *Q. nuttallii* and *Q. palustris* before and after flooding treatment. (**A**) L*, lightness—positive toward white and negative toward black. (**B**) a*, red–green value—positive toward red and negative toward green. (**C**) b*, yellow–blue value—positive toward yellow and negative toward blue. (**D**) a/b, the ratio of red–green value to yellow–blue value. (**E**) C*, chroma value. (**F**) ΔE, the overall color difference. Columns with different lowercase letters are statistically different (*p* < 0.05).

For *Q. nuttallii*, all tested color parameters were increased by flooding, while for *Q. palustris*, no differences in b* and C* values were observed between flooding and control conditions or between days 0 and 60 (Figure 2). The ΔE value of *Q. palustris* was smaller than that of *Q. nattallii* under both control and flooding conditions (Figure 2E), suggesting that *Q. palustris* has less color variation and higher color stability.

### 2.2. The Impact of Flooding on Gas Exchange and Chlorophyll Fluorescence Parameters

**Figure 3:**
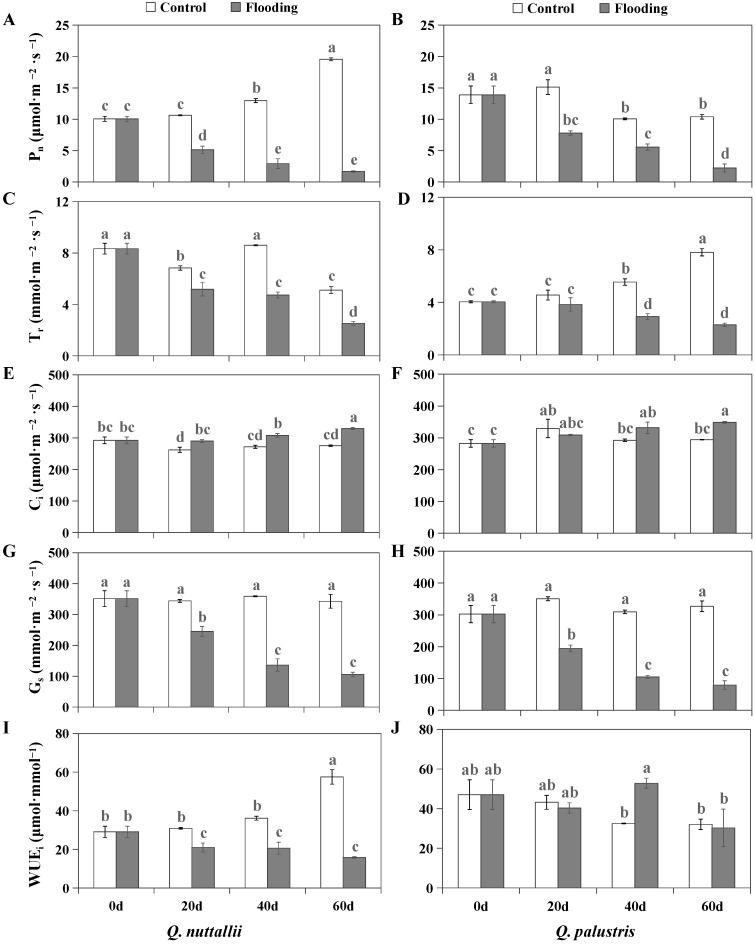
Gas exchange parameters, including P_n_, T_r_, G_s_, C_i_, and WUE_i_, of *Q. nuttallii* (**A**,**C**,**E**,**G**,**I**) and *Q. palustris* (**B**,**D**,**F**,**H**,**J**) seedlings in the treatment days under flooding stress. (**A**,**B**) P_n_, maximum net photosynthetic rate. (**C**,**D**) T_r_, transpiration rate. (**E**,**F**) C_i_, intercellular CO_2_ concentration. (**G**,**H**) G_s_, stomatal conductance. (**I**,**J**) WUE_i,_ instantaneous water use efficiency. Data are given as means ± SE (n = 3). Columns with different lowercase letters are statistically different (*p* < 0.05).

Flooding treatment significantly increased *C*_i_ and decreased P_n_, T_r_, and G_s_ in both *Q. nuttallii* and *Q. palustris* (Figure 3A–H). In addition, the gas exchange parameters were significantly affected by species, treatment, and days. However, there was no significant effect of species × treatment × time on G_s_ and C_i_ (Table 2). Under control conditions, *Q. nuttallii* had higher values of P_n_ and WUE_i_ (Figure 3A,B,I,J). Compared with their controls, greater decreases in P_n_ and WUE_i_ were found for *Q. nuttallii* than for *Q. palustris* under flooding conditions (Table 3). In other words, flooding caused greater inhibition of P_n_ and WUE_i_ in *Q. nuttallii* than in *Q. palustris*. 

**Figure 4:**
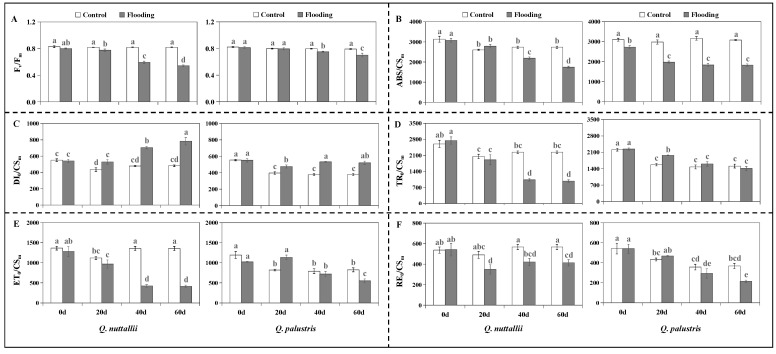
Chlorophyll fluorescence parameters including F_v_/F_m_ (**A**), ABS/CS_m_ (**B**), DI_0_/CS_m_ (**C**), TR_0_/CS_m_ (**D**), ET_0_/CS_m_ (**E**), and RE_0_/CS_m_ (**F**) of *Q. nuttallii* (left figures) and *Q. palustris* (right figures) seedlings in the treatment days under flooding stress. Data are given as means ± SD (n = 3). Columns with different lowercase letters are statistically different (*p* < 0.05).

Chlorophyll fluorescence parameters including F_v_/F_m_, ABS/CS_m_, TR_0_/CS_m_, ET_0_/CS_m_, and RE_0_/CS_m_ in both *Q. nuttallii* and *Q. palustris* were significantly decreased after the flooding treatment (Figure 4A,B,D–F). TR_0_/CS_m_, ET_0_/CS_m_, and RE_0_/CS_m_ were not significantly affected in terms of species. All tested chlorophyll fluorescence parameters were significantly affected by the water treatments and treatment days, while RE_0_/CS_m_ was not significantly affected by the interaction of species × treatment × time (Table 2). Under control conditions, *Q. nuttallii* had a lower ABS/CS_m_ value than *Q. palustris*, whereas *Q. palustris* had lower DI_0_/CS_m_, TR_0_/CS_m_, ET_0_/CS_m_, and RE_0_/CS_m_ values (Figure 4B–F). Compared with the controls, *Q. nuttallii* exhibited greater decreases in F_v_/F_m_, TR_0_/CS_m_, ET_0_/CS_m_, and RE_0_/CS_m_ than *Q. palustris* under flooding conditions. These results indicate that photosynthetic performance was more impaired in *Q. nuttallii* than in *Q. palustris*.

### 2.3. The Impact of Flooding on Biochemical Parameters in Both Oak Species

**Figure 5:**
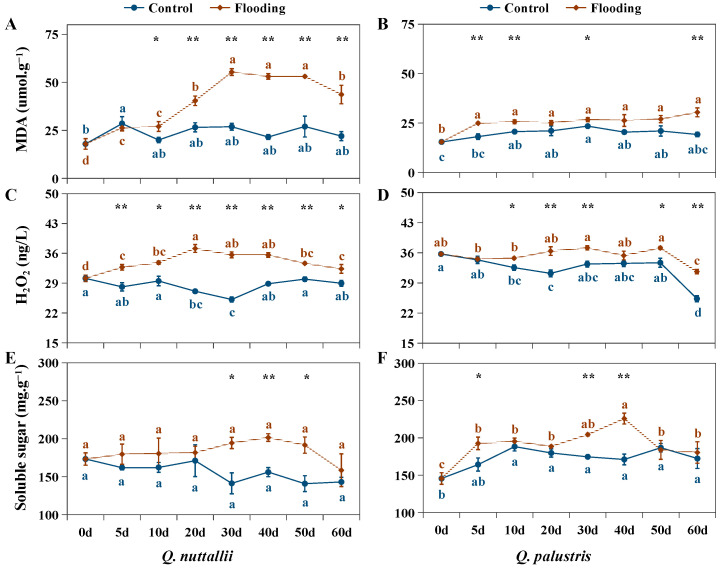
MDA (**A**,**B**), H_2_O_2_ (**C**,**D**), and soluble sugar (**E**,**F**) content in *Q. nuttallii* (**A**,**C**,**E**) and *Q. palustris* (**B**,**D**,**F**) seedlings in the treatment days under flooding stress. Data are given as means ± SD (n = 3). Polylines with different lowercase letters are statistically different (*p* < 0.05). The results of *t*-test that discerning difference between the two treatments was labelled by ‘*’ (*p* < 0.05) and ‘**’ (*p* < 0.01) above the letters.

**Figure 6:**
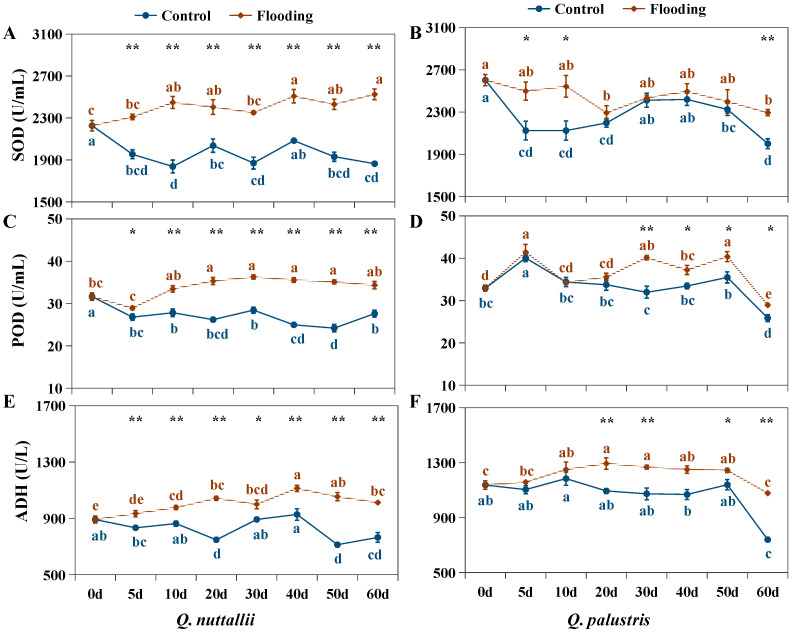
Content of SOD (**A**,**B**), POD (**C**,**D**), and ADH (**E**,**F**) of *Q. nuttallii* (**A**,**C**,**E**) and *Q. palustris* (**B**,**D**,**F**) seedlings in the treatment days under flooding stress. Data are given as means ± SD (n = 3). Polylines with different lowercase letters are statistically different (*p* < 0.05). The results of *t*-test that discerning difference between the two treatments was labelled by ‘*’ (*p* < 0.05) and ‘**’ (*p* < 0.01) above the letters.

Flooding treatment caused significant increases in MDA, H_2_O_2_, SOD, POD, and ADH in both *Q. nuttallii* and *Q. palustris* (Figure 5 and Figure 6), and the parameters were significantly affected by the interaction of species × treatment × time (Table 2). POD was activated from day 30 and ADH was activated from day 20 in *Q. palustris*, i.e., later than in *Q. nuttallii* (Figure 6C–F). Unlike in *Q. nuttallii*, the physiological parameters of *Q. palustris* in the middle of the stress phase (days 20 to 50) were not higher than those of the control group throughout the entire stage, as in *Q. nuttallii* (Figure 5B,D,F and Figure 6B,D,F). These results indicate that the activation of the antioxidant defense system occurred earlier in *Q. nuttallii* than in *Q. palustris*.

Although the defense response was activated in both *Q. nuttallii* and *Q. palustris*, the increase in MDA and H_2_O_2_ content was higher under flooding conditions than under control conditions. However, the fewest changes in antioxidant enzymes and osmotic substances were observed in *Q. palustris* between the flooding and control treatments in the middle of the flooding period (Figure 5A–D, Table 4 and Table 5). Compared with their controls, the MDA content was higher in *Q. nuttallii* than in *Q. palustris* on these treatment days. The soluble sugar content was also higher in *Q. nuttallii* than in *Q. palustris* in the middle of the flooding period (Table 4 and Table 5). The H_2_O_2_ and ADH proportions in *Q. nuttallii* were higher at the beginning and middle of the flooding period and lower at the end (days 50 to 60) than in *Q. palustris*. The H_2_O_2_, SOD, POD, and ADH content in the leaves decreased in *Q. palustris* at the end of the treatment period (days 50 to 60) under both control and flooding conditions (Figure 5D and Figure 6B,D,F). This was not observed in *Q. nuttallii*. During the treatment periods, the SOD and POD proportions in the leaves were higher in *Q. nuttallii* than in *Q. palustris* (Figure 6A–D). However, the MDA and H_2_O_2_ proportions were also higher in *Q. nuttallii*, suggesting that *Q. nuttallii* was more sensitive to flooding stress. 

### 2.4. The Impact of Flooding on Endogenous Hormone Levels

**Figure 7:**
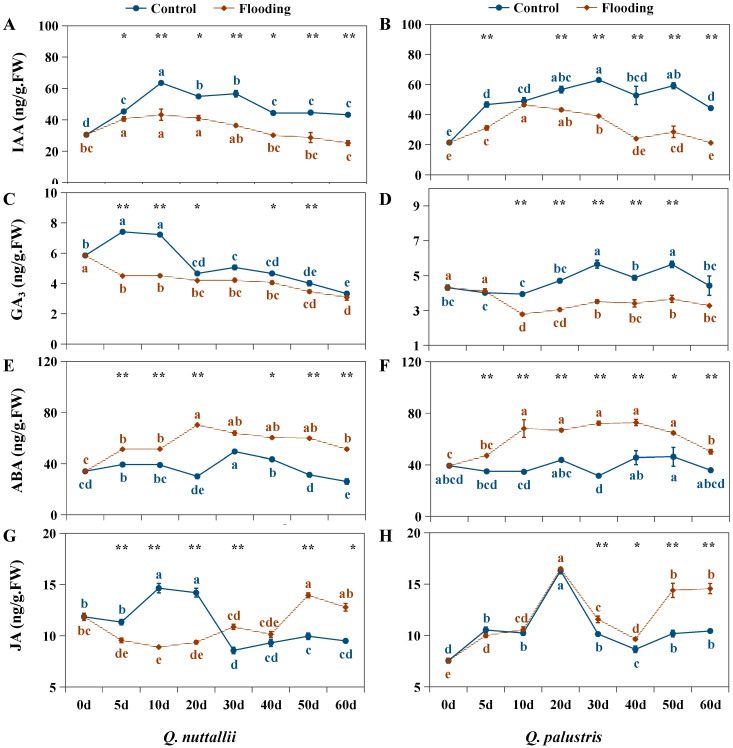
Content of IAA (**A**,**B**), GA_3_ (**C**,**D**), ABA (**E**,**F**), and JA (**G**,**H**) in *Q. nuttallii* (**A**,**C**,**E**,**G**) and *Q. palustris* (**B**,**D**,**F**,**H**) seedlings in the treatment days under flooding stress. Data are given as means ± SD (n = 3). Polylines with different lowercase letters are statistically different (*p* < 0.05). The results of *t*-test that discerning difference between the two treatments was labelled by ‘*’ (*p* < 0.05) and ‘**’ (*p* < 0.01) above the letters.

Flooding treatment significantly decreased IAA levels and increased ABA and JA levels in both *Q. nuttallii* and *Q. palustris*. GA_3_ levels were decreased on day 50, and there was no difference between flooding and control conditions on day 60 (Figure 7). The four hormones were significantly affected by the interaction of species × treatment × time (Table 2). IAA levels increased and peaked on day 10 in both *Q. nuttallii* and *Q. palustris* (Figure 7A,C), while they were lower in the middle and at the end of the flooding treatment. In Q. palustris, the GA_3_ level decreased and reached the valley value on day 10 (Table 4 and Table 5). Both ABA and JA levels were higher in *Q. palustris* than in *Q. nuttallii* in the early period of the flooding treatments (Figure 7E–H, Table 4 and Table 5). The JA content was also higher in *Q. palustris* in the middle and late periods (Table 5). In *Q. nuttallii*, the pattern of the ABA content was like that in *Q. palustris* (Figure 7E,F). Compared with the control conditions, the JA content decreased and then increased under flooding conditions (Figure 7G). 

### 2.5. Pearson Correlation Coefficients (PCCs) and Principal Component Analysis (PCA) of Physiological Parameters under Flooding Stress

**Figure 8:**
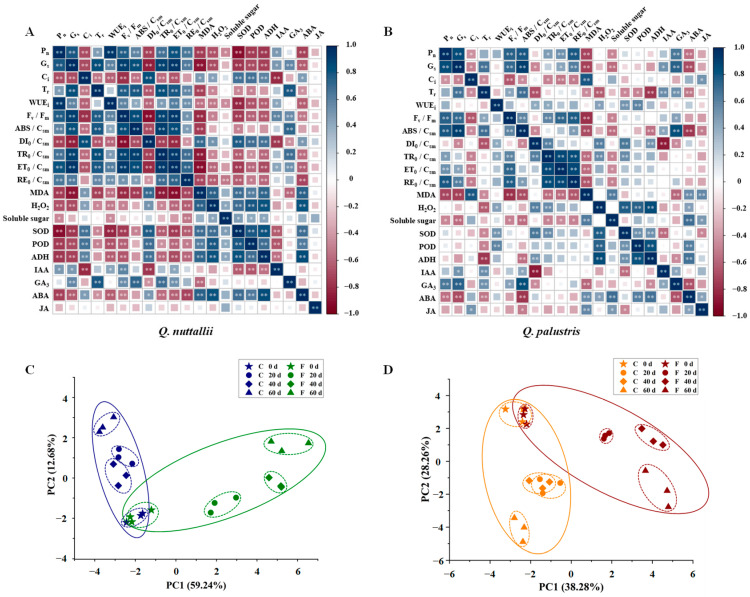
Pearson correlation analysis (**A**,**B**) and principal component analysis (**C**,**D**) of photosynthetic and physiological parameters of *Q. nuttallii* (**A**,**C**) and *Q. palustris* (**B**,**D**) seedlings in the treatment days under flooding stress on days 0, 20, 40, and 60. The results of the pearson correlation analysis that discerning the difference between the two parameters were labeled by ‘*’ (*p* < 0.05) and ‘**’ (*p* < 0.01) above the letters.

PCCs and PCA were derived and performed, respectively, based on gas exchange parameters, chlorophyll fluorescence parameters, biochemical parameters, and endogenous hormones to test whether the composition of the physiological parameters of *Q. nuttallii* and *Q. palustris* leaves differed between the flooding treatments and different days (Figure 8). The heat maps of the PCCs show significant correlations for 21 parameters in both *Q. nuttallii* (Figure 8A) and *Q. palustris* (Figure 8B). 

In *Q. nuttallii*, WUE_i_ was correlated with P_n_ (PCC = 0.95), and G_s_ was correlated with P_n_ (PCC = 0.80), F_v_/F_m_ (PCC = 0.91), ABS/CS_m_ (PCC = 0.84), TR_0_/CS_m_ (PCC = 0.89), and ET_0_/CS_m_ (PCC = 0.95). ADH was correlated with H_2_O_2_ (PCC = 0.80), SOD (PCC = 0.82), and ABA (PCC = 0.85) (Figure 8A and Table S1). PC1 and PC2 accounted for 59.24% and 12.68% of the total variation in *Q.nuttallii*, respectively (Figure 8C). The samples under two water conditions were almost divided into two clusters by PC1 (Figure 8C); they were affected by P_n_, G_s_, F_v_/F_m_, TR_0_/CS_m_, ET_0_/CS_m_, MDA, SOD, and ADH. PC2 was influenced by T_r_, GA_3_, and ABS/CS_m_ (Table S3).

In *Q. palustris*, G_s_ was correlated with P_n_ (PCC = 0.84) and ABS/CS_m_ (PCC = 0.91). ADH was correlated with H_2_O_2_ (PCC = 0.84) and POD (PCC = 0.80) (Figure 8B and Table S2). PC1 and PC2 accounted for 38.28% and 28.26% of the total variation in *Q. palustris*, respectively (Figure 8D). Of all the parameters tested, P_n_, G_s_, F_v_/F_m_, ABS/CS_m_, RE_0_/CS_m_, and MDA contributed the most to PC1 when clustering two different water conditions (Table S4). PC2 was influenced by DI_0_/CS_m_, TR_0_/CS_m_, H_2_O_2_, and ADH (Table S4).

## 3. Discussion

### 3.1. The Two Species Had Contrasting Growth Responses

In general, flooding tolerance is evaluated in terms of the growth response of trees, the level of injury sustained, and survival with respect to flooding level (depth) and duration [25,26,27]. The present study detected different adaptive capabilities between *Q. nuttallii* and *Q. palustris* under flooding conditions. All treated plants could survive after 60 days of flooding treatment leading to water up to 5 cm above the soil surface, indicating that these two oak species possessed some tolerance to flooding.

Although *Q. nuttallii* exhibited greater growth under control conditions, flooding inhibited seedling growth more in *Q. nuttallii* than in *Q. palustris* (Figure 1C,D). This result agrees with results from previous studies and suggests that morphology can be altered to adapt to flooding [28,29]. The two analyzed species developed morphological plasticity to tolerate flooding conditions. *Q. nuttallii* was more responsive to flooding stress. In addition, stem hypertrophy was an adaptive response to flooding, a primary pathway for atmospheric air transmission to the roots for aeration [30,31]. Changes in stem diameter are closely correlated with plants’ flooding tolerance [31,32]. The relationship between stem development and flooding tolerance could be useful in selecting flood-tolerant *Quercus* species, which can be based on morphological adaptations to different conditions [31]. 

Flooding reduced the gas exchange parameters (P_n_, T_r_, and G_s_) but increased C_i_ in both *Quercus* species (*Q. nuttallii* and *Q. palustris*) (Figure 3 and Table 3). These results indicate that the reduction in G_s_ affected the absorption of CO_2_, allowing the CO_2_ produced via respiration to accumulate in the leaves. Stomatal opening decreased sharply to reduce water loss in response to flooding stress, while P_n_ and T_r_ were inhibited by stomatal closure [33,34]. This was because the root system was first affected by flooding, leading to changes in secondary metabolism and limited water uptake and transport, and then photosynthesis was suppressed [35]. In this study, G_s_ was correlated with P_n_ in both *Q. nuttallii* (PCC = 0.80) and *Q. palustris* (PCC = 0.80). In addition, P_n_, T_r_, and G_s_ showed consistent trends, and the ABA concentration was elevated to regulate stomatal closure under flooding stress in the two species. These results suggest that the decreases in P_n_ and T_r_ were largely due to stomatal limitations. This could be due to the accumulation of non-structural carbohydrates in the leaves causing feedback inhibition of photosynthesis [29,36]. Greater reductions in the WUE_i_ were found in *Q. nuttallii* than in *Q. palustris* in response to flooding stress. This suggests that in contrast to *Q. nuttallii*, *Q. palustris* has more efficient endogenous defensive mechanisms for alleviating photosynthetic damages in response to flooding stress.

In this study, F_v_/F_m_ was lower in both *Quercus* species under flooding conditions on day 60 than in the control (Figure 4A and Table 3), suggesting that photosynthetic metabolism was impaired and reaction centers were damaged by flooding. The findings of this study support our first hypothesis that flooding stress would inhibit plant growth and leaf photosynthesis and decrease stomatal conductance and F_v_/F_m_. In addition, the main chlorophyll fluorescence parameters including phenomenological fluxes (ABS/CS_m_, TR_0_/CS_m_, ET_0_/CS_m_, and RE_0_/CS_m_) were limited by flooding [37,38]. Stomatal closure resulted in the limitation of carbon dioxide (CO_2_) diffusion to the chloroplasts in addition to net CO_2_ assimilation [39,40]. The alternative energy dissipation mechanisms, including heat exchange and photorespiration, were triggered by the limited utilization of the decreasing energy during CO_2_ reduction [33,40]. These results indicate that photoinhibition caused by flooding stress occurred in both *Q. nuttallii* and *Q. palustris*, and the defense mechanisms were initiated in both species to dissipate the excess excitation energy. It has been reported that another reason for a reduction in ET_0_/CS_m_ is blocked electron transport, which could be due to the inhibition of the oxygen release cycle (OEC) and energy absorption of the antenna pigments [38,41,42]. The greater decrease in ABS/CS_m_ in *Q. palustris* indicates a limitation of light absorption (Figure 4B), which could be a defense response of *Q. palustris* under flooding conditions [43,44]. These results show that *Q. nuttallii* and *Q. palustris* exhibited different photosynthetic patterns under flooding and control conditions, respectively. *Q. nuttallii* exhibited a higher photosynthetic capacity under control conditions, while *Q. palustris* demonstrated less photosynthetic inhibition under flooding stress.

### 3.2. Higher Tolerance and Lower Sensitivity Were Observed in Q. palustris

*Q. nuttallii* and *Q. palustris* showed similar response patterns in antioxidant defense and osmotic adjustment to flooding stress, while their tolerance and sensitivity to flooding were different. PC1 and PC2 of *Q. nuttallii* and *Q. palustris* were affected by P_n_, G_s_, F_v_/F_m_, ABS/CS_m_, MDA, and ADH, indicating the similarities between both species in their adaptive changes under flooding treatments. Most terrestrial plants cannot tolerate prolonged waterlogging stress. Plants may be killed by toxic accumulation, C deficiency, cytoplasmic acidification, or disease under continuous or repeated flooding conditions. The earlier the flood, the more sensitive the species [45]. Wetland species, including oaks, can minimize or postpone physiological stress to undergo adaptive changes during flooding [40]. The significant changes in P_n_, T_r_, G_s_, WUE_i_, F_v_/F_m_, TR_0_/CS_m_, and ET_0_/CS_m_ indicate that *Q. nuttallii* is more sensitive to flooding stress than *Q. palustris* in terms of photosynthesis and chlorophyll fluorescence. *Q. nuttallii* was also more responsive and showed greater negative phenotypic effects when grown under flooding conditions. 

The root hypoxia induced by the flooding stress caused photo-oxidative damage to the leaves by increasing the production of ROS [26,46]. As important signaling molecules that can directly attack membrane lipids, ROS lead to lipid peroxidation and the oxidation of proteins and nucleic acids [27,47]. The degree of lipid peroxidation in the membranes was reflected in the MDA content, one of the most commonly used indicators of lipid peroxidation [27,46]. Although the activation of the antioxidant defense system occurred earlier in *Q. nuttallii* than in *Q. palustris* (Figure 6, Table 3 and Table 4), the MDA and H_2_O_2_ levels were higher in *Q. nuttallii* than in *Q. palustris* in the middle and end of the flooding period (Figure 5, Table 3 and Table 4), indicating that *Q. nuttallii* plants were exposed to more severe oxidative damage during the growth period, while *Q. palustris* reduced and delayed the stress via adaptative changes compared with *Q. nuttallii*. 

Plants have a set of scavenging enzymes that help them cope with ROS, such as SOD and POD [27,47]. SOD catalyzes O_2_ to H_2_O_2_ and gaseous oxygen, and POD and other antioxidant enzymes decompose H_2_O_2_ [47]. In this study, both *Q. nuttallii* and *Q. palustris* exhibited higher SOD and POD activities in response to flooding stress compared with the individual controls. This suggests that these species are equipped with efficient antioxidant systems that protect them from oxidative damage caused by flooding [48,49,50]. In addition, the lower MDA, H_2_O_2_, and POD levels in *Q. palustris* in response to flooding stress suggests that *Q. palustris* possesses a better ability to maintain the balance between the formation and detoxification of activated oxygen species and possesses better capacities to protect itself from severe oxidative damage [51,52]. 

Both *Q. nuttallii* and *Q. palustris* can maintain higher levels of soluble sugars during the middle period of flooding. It has been reported that plants can keep greater levels of soluble sugars under stress, and the observed increase in soluble sugars indicates that osmotic regulation is likely a strategy for plants to cope with stress [29,53]. However, the accumulation of soluble sugars was observed earlier in *Q. palustris* than in *Q. nuttallii*, suggesting *Q. palustris* contains a greater metabolic energy supply to respond to flooding stress. There is clear experimental evidence that more tolerant species maintain carbohydrate concentrations at a high level [10,54,55,56,57]. In this study, *Q. palustris* exhibited higher soluble sugar content than *Q. nuttallii* under flooding and control conditions. Additionally, both ABA and JA levels were higher in *Q. palustris* than in *Q. nuttallii* in the early period of the flooding treatments (Figure 7E–H, Table 4 and Table 5). It is widely reported that ABA, SA, and JA participate in the antioxidant defense against abiotic and biotic stresses, playing critical roles in stress tolerance by regulating the antioxidant defense system, reducing ROS levels, and alleviating the degradation of internal cell organelles [58,59,60,61]. Therefore, increased levels of ABA and JA may benefit flooding tolerance in the early period with respect to both enzymatic (SOD) and non-enzymatic (soluble sugars) facets in *Q. palustris* [58,59]. Further analyses at the physiological and molecular level are needed to clarify the mechanisms of phytohormone regulation [62,63]. 

*Q. nuttallii* responded earlier to flooding stress than *Q. palustris* by regulating stomata and antioxidant defenses, which was attributed to the increase in the ABA levels of antioxidant enzymes. The lower degree of photosynthetic inhibition in *Q. palustris* could be related to the plants’ lower sensitivity. The degree of inhibition via flooding was lower in *Q. palustris* than in *Q. nuttallii* for all measured parameters. *Q. nuttallii* was confirmed to be more sensitive to stress. The regulating effect of *Q. palustris* materialized in the later stages of flooding stress. 

This study only studied the flood response for two oak species. Therefore, the possibility of generalizing these results to other species or environmental conditions may be limited. Additionally, we only examined short-term responses to flooding. If the experiment lasted much longer, the trait responses could be more significant. The seedlings were potentially more susceptible to flooding stress than the adult trees. Therefore, the results may differ from those regarding adult trees in the field, and the characteristic responses in adult trees may be less intense. Despite these limitations, this short-term study showed that seedlings of *Q. nuttallii* and *Q. palustris* respond differently to different flooding periods, which may improve the physiological understanding of the ability of seedlings to cope with flooding stress. With more frequent flooding, plant resistance and resilience to flooding in agricultural and forestry environments should be further explored, as our study was conducted from the perspective of a holobiont [40].

## 4. Materials and Methods

### 4.1. Study Area

This experiment was conducted at an experimental site of the National Repository of Crabapple Germplasm in Yangzhou City, China (32°42′ N, 119°55′ E). This area has a northern subtropical monsoon climate, with an average annual temperature of 14.9 °C and a precipitation amount of 1000 mm. It has 320 frost-free days, a flat terrain, and sandy loam, which is deep and fertile, alongside favorable irrigation and drainage conditions.

### 4.2. Plant Material and Experimental Design

A pot experiment with a factorial design with respect to flooding treatment and species was carried out during the growing season. Two oak species, *Quercus nuttallii* and *Quercus palustris*, were used. In fall 2017, acorns of *Q. nuttallii* and *Q. palustris* were collected from the parent trees at the National Repository of Crabapple Germplasm (32°42′ N, 119°55′ E) and overwintered at 4 °C. In spring 2018, the acorns were sown in a plastic case (2 m long × 1.4 m wide × 0.45 m deep) and grown in a growth chamber (average temperature: 25 °C; relative humidity: 60–70%; light: 16 h per day). In January 2019, the seedlings were transplanted into the pots and grown on the trial site. The pots were non-woven fabric bags with a diameter of 25 cm and a height of 30 cm, filled with matrix, vermiculite, and perlite (matrix–vermiculite–perlite 2:1:1, *v*/*v*). In each pot, 2 seedlings were cultivated for 6 months under normal irrigation and outdoor conditions until they had reached a stable state. In July 2019, the seedlings of *Q. nuttallii* and *Q. palustris* with uniform size were selected, and the height of the seedlings was approximately 40 cm. 

For the experimental design, two species (*Q. nuttallii* and *Q. palustris*) were combined with two treatments (flooding and control), resulting in a combination of 4 treatments in this study. Of the total of 96 seedlings obtained after adaptive cultivation, 48 seedlings of *Q. nuttallii* or *Q. Palustris* were selected and divided into two groups: the control group (approximately 75% of field water capacity) and the flooding treatment group (wherein the soil water content was supersaturated, and flooding exceeded the soil surface by 5 cm). Each combination contained 24 seedlings, which were harvested 8 times (on days 0, 5, 10, 20, 30, 40, 50, and 60, respectively), with 3 seedlings harvested each time. The flooding treatment was performed on the 180th day after transplanting and lasted for 60 days. The seedlings were transferred with the pots into the tank (height 5 cm) to simulate artificial flooding and maintain long-term flooding conditions. The water tank contained both seedlings and pots, in which the seedlings remained in the pots. Flooding occurred in the tank, the surface of which remained 5 cm above the soil (in the pots) and was replaced when necessary. The soil in the tank was sandy loam, and the seedlings were not shaded by the walls. The planting area of each treatment unit was 8 m^2^ (8 m long and 1 m wide), and the soil thickness was 30 cm.

### 4.3. Measurement of Gas Exchange and Chlorophyll Fluorescence Parameters

Gas exchange was monitored using the portable photosynthetic device CIRAS-2 (PP Systems, London, UK) on the mornings of days 0, 20, 40, and 60 (8:00−11:00) during the flooding treatment. The light intensity was set to 1000 μmol·m^−2^·s^−1^, the CO_2_ concentration in the reference chamber was 380 μmol·mol^−1^, and the leaf temperature was 25 °C. The fully functional leaves were measured at the 3rd–5th nodes of the upper part of the plantlet, obtaining the average growth level for three replicated seedlings in each treatment. The net photosynthetic rate (P_n_), stomatal conductance (G_s_), intercellular CO_2_ concentration (C_i_), and transpiration rate (Tr) of the leaves were recorded. The instantaneous water use efficiency (WUE_i_) was calculated according to the ratio of P_n_ to G_s_.

The chlorophyll fluorescence parameters were measured using a Handy PEA continuous fluorometer (Hansatech, London, UK) at 8:00−10:00 a.m. on days 0, 20, 40, and 60 after flooding. The 10 functional leaves from the 4th to 7th position of the upper part of the plantlets were selected and adjusted to darkness for 30 min. The time difference of leaf adaptation was 2 min. After a certain time, the basic fluorescence parameters were determined with a light intensity of 3000 μmol·m^−2^·s^−1^. The OJIP kinetics were measured in the first fully expanded youngest leaf approximately 1 h after the chamber was switched off. Chlorophyll fluorescence was measured in approximately one-third of the leaf tip, avoiding the leaf veins. For each measurement, a detachable leaf clip was placed on a leaf, and the fluorescence probe was positioned perpendicular to the surface of the leaf clip. The fluorescence signal was displayed with a temporal resolution of 10 μs. At the beginning of the measurement, weak light of 2–3 μmol quantum m^−2^ s^−1^ was irradiated onto the upper epidermis. A saturated light pulse of 3500 μmol quantum m^−2^ s^−1^ was emitted with a peak wavelength of 627 nm for 1 s [64].

The measured parameters included the absorption flux per unit area (ABS/CS_m_), the energy per unit area used for heat dissipation (DI_0_/CS_m_), the trapped energy flux per unit area (TR_0_/CS_m_), the energy per unit area used for electron transport (ET_0_/CS_m_), the number of reaction centers per unit area (RE_0_/CS_m_), and the maximum efficiency of PSII (F_v_/F_m_).

### 4.4. Measurements of Seedling Growth Traits and Leaf Color

Seedling growth characteristics, including plant height and diameter, were determined on days 0 and 60, respectively. The height from the soil surface to the apical bud was measured on the main stem. The diameter was determined 10 cm above the soil surface using a micrometer caliper. The leaves of *Q. nuttallii* and *Q. palustris* of both the control and treatment groups were collected and photographed. The leaves and the x-rite 24-color calibration card were tiled on the same white plate and photographed with the same camera (Canon EOS5DSR, Tokyo, Japan) under the same conditions. The aperture value was f/16, the exposure time was 1/160 s, and the flash was forced. The colorfulness of the leaves was recorded in CIELAB parameters, including the lightness value (L*), the red–green value (a*), the yellow–blue value (b*), the chroma value (C*), the total color difference (ΔE), and the a*/b* value [65,66]. 

### 4.5. Harvest and Measurements of MDA, H_2_O_2,_ and Soluble Sugar

The leaves of *Q. nuttallii* and *Q. palustris* were harvested at 10:00 a.m. on days 0, 5, 10, 20, 30, 40, 50, and 60 in the control and treatment groups. The harvested leaves were wrapped in aluminum foil and immediately frozen in liquid nitrogen for 1 h. The frozen samples were then ground into fine powder in liquid nitrogen and stored at −80 °C for later determination. 

The malondialdehyde (MDA) concentration was determined using 0.3 g of fresh leaf samples extracted at 4 °C with 5 mL of 0.05 M PBS (pH 7.8). Then, the extracts were mixed with 2.5 mL of thiobarbituric acid and heated at 100 °C for 15 min. After cooling in the ice, the samples were centrifuged at 1800 r/min for 10 min and spectrophotometrically measured at wavelengths of 450 nm, 532 nm, and 600 nm [67]. The hydrogen peroxide (H_2_O_2_) concentration was spectrophotometrically determined at 505 nm using the 4-aminoantipyrine method, as previously described by Alvarez et al. (2009) [68]. The soluble sugar content was measured using the Anthrone method described by Shi et al. (2017) [69].

### 4.6. Analysis of Enzyme Activities and Endogenous Hormone Levels

The antioxidant enzymes analyzed included superoxide dismutase (SOD; EC 1.15.1.1) and peroxidase (POD; EC 1.11.1.7), measured in the leaves using a method detailed in previous reports [62]. One unit of SOD was defined as the amount of enzyme causing half the maximum inhibition of NBT reduction [70,71,72]. One unit of POD was defined as the amount of enzyme that oxidized 1 mmol of guaiacol min^−1^ mg^−1^ protein and was determined spectrophotometrically at a wavelength of 290 nm. The alcohol dehydrogenase (ADH) activity was determined according to the method described by Yin et al. (2009) [47]. One unit of ADH was defined as the amount of enzyme that reduced the substrate per minute and was determined spectrophotometrically at a wavelength of 340 nm. 

The endogenous hormones analyzed included indole-3-acetic acid (IAA), gibberellic acid (GA_3_), jasmonic acid (JA), and abscisic acid (ABA). Over 0.5 g of each sample was collected and analyzed using the enzyme-linked immunosorbent assay (ELISA) method. The College of Agronomy and Biotechnology, China Agricultural University, provided the kit used for endogenous hormone determination.

### 4.7. Statistical Analysis

Statistical analysis was performed using SAS 8.4 software, and the normality of all data was tested using the UNIVARIATE program in SAS (SAS Institute, Cary, NC, USA; 1996). To detect the differences and interactions of species, treatment, and time for experimental variables, three-way ANOVA and multiple mean comparisons (Duncan’s test) were applied. A one-way ANOVA was used to compare the differences in photosynthetic and physiological parameters between the control and flooding conditions for both *Q. nuttallii* and *Q. palustris*. A *t*-test was used to compare the differences between the control and flooding conditions on the same treatment days for *Q. nuttallii* and *Q. palustris*, respectively. Differences were considered significant when *p* ≤ 0.05. After the data were standardized, principal component analysis (PCA) and correlation analysis were performed using R-3.6.2 software (http://www.rproject.org/, accessed on 1 October 2023) and plotted using Origin 2021 software (Origin Lab Corporation, Northampton, MA, USA).

## 5. Conclusions

**Figure 9:**
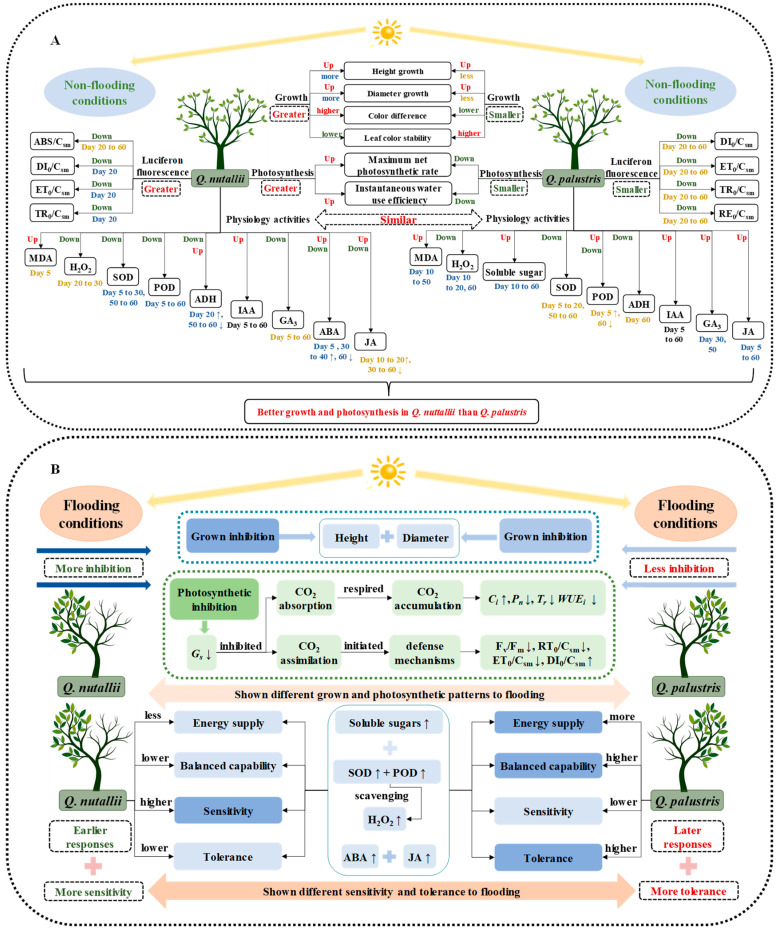
The schematic model of the physiological responses of *Q. nuttallii* and *Q. palustris* seedlings in different periods under control conditions and flooding stress. (**A**) The physiological schematic model of *Q. nuttallii* and *Q. palustris* under control conditions. (**B**) The physiological schematic model of *Q. nuttallii* and *Q. palustris* under flooding stress. ‘↑’, significantly increased. ‘↓’, significantly decreased. ‘ns’, not significant.

A schematic model of the morphological, photosynthetic, and physiological responses of seedlings of *Q. nuttallii* and *Q. palustris* seedlings under non-flooding and flooding conditions is presented (Figure 9). The growth and photosynthetic responses of *Q. nuttallii* and *Q. palustris* under flooding and natural conditions differed, respectively. *Q. nuttallii* grew more vigorously and had a higher photosynthetic capacity under control conditions, while *Q. palustris* was less inhibited under flooding stress. The decreases in P_n_ and T_r_ were largely due to stomatal limitations. The greater decrease in ABS/CS_m_ in *Q. palustris* indicates the limitation of light absorption, which could be a defense response of *Q. palustris* under flooding conditions. The species exhibited similar response patterns in their antioxidant defenses to flooding stress, whereas their tolerance and sensitivity to flooding differed. The higher tolerance and lower sensitivity of *Q. palustris* to flooding were associated with an enhanced ability to maintain photosynthesis (WUEi) and antioxidant defense systems, combined with fewer morphological (growth traits) and physiological (light absorption, photoinhibition, and phytohormone homeostasis) restrictions. The lower tolerance of *Q. nuttallii* to flooding was associated with greater accumulations of MDA, H_2_O_2_, and soluble sugars. *Q. nuttallii* plants underwent more severe oxidative damage during the growing season, while *Q. palustris* reduced and delayed stress via adaptative changes more than *Q. nuttallii*.

## Figures and Tables

**Table 1:** The impact of flooding on plant growth and leaf color.

Phenotypic Parameters	*Q. nuttallii* (Day 60)	*Q. palustris* (Day 60)
Height (growth amount)	0.32 ↓	0.67 ↓
Diameter (growth amount)	0.48 ↓	0.63 ↓
ΔE	1.82 ↑	2.71 ↑
L*	1.50 ↑	1.23 ↑
a*	2.00 ↑	1.83 ↑
b*	1.81 ↑	0.94 ns
a/b	3.62 ↑	1.70 ↑
C*	1.53 ↑	0.87 ns

Note: ‘↑’, significantly increased. ‘↓’, significantly decreased. ‘ns’, not significant.

**Table 2:** Means and interaction effects of species and treatment times.

	Indexes	Species	Treatment	Time	Species × Treatment	Species × Time	Treatment × Time	Species × Treatment × Time
1	P_n_	*	**	**	**	**	**	**
2	G_s_	**	**	**	Ns	Ns	**	Ns
3	C_i_	**	**	*	Ns	*	**	Ns
4	T_r_	**	**	**	Ns	**	**	**
5	WUE_i_	**	**	Ns	**	**	**	**
6	F_v_/F_m_	**	**	**	**	**	**	**
7	ABS/CS_m_	Ns	**	**	**	**	**	**
8	DI_0_/CS_m_	**	**	**	**	**	**	*
9	TR_0_/CS_m_	**	**	**	**	Ns	**	**
10	ET_0_/CS_m_	**	**	**	**	Ns	**	**
11	RE_0_/CS_m_	**	**	**	Ns	**	*	Ns
12	MDA	**	**	**	**	**	**	**
13	H_2_O_2_	**	**	**	**	**	**	**
14	Soluble sugar	**	**	**	Ns	*	*	Ns
15	SOD	**	**	**	**	**	**	*
16	POD	**	**	**	**	**	**	*
17	ADH	**	**	**	Ns	**	**	**
18	IAA	Ns	**	**	*	**	**	**
19	GA_3_	**	**	**	Ns	**	**	**
20	ABA	**	**	**	Ns	**	**	**
21	JA	Ns	**	**	**	**	**	**

Note: ‘*’, significant difference (*p* < 0.05). ‘**’, significant difference (*p* < 0.01). ‘Ns’, not significant.

**Table 3:** The impact of flooding on gas exchange and chlorophyll fluorescence parameters.

Photosynthetic Indexes	*Q. nuttallii*			*Q. palustris*		
	20 d	40 d	60 d	20 d	40 d	60 d
P_n_	0.48 ↓	0.23 ↓	0.09 ↓	0.52 ↓	0.55 ↓	0.21 ↓
G_s_	0.71 ↓	0.38 ↓	0.31 ↓	0.56 ↓	0.34 ↓	0.24 ↓
C_i_	1.11 ↑	1.13 ↑	1.20 ↑	0.94 ns	1.13 ns	1.18 ↑
T_r_	0.76 ↓	0.55 ↓	0.49 ↓	0.84 ns	0.53 ↓	0.29 ↓
WUE_i_	0.64 ↓	0.40 ↓	0.17 ↓	0.62 ↓	1.05 ns	0.71 ns
F_v_/F_m_	0.95 ↓	0.73 ↓	0.66 ↓	1.00 ns	0.94 ↓	0.88 ↓
ABS/CS_m_	1.07 ns	0.80 ↓	0.64 ↓	0.66 ↓	0.58 ↓	0.59 ↓
DI_0_/CS_m_	1.22 ↑	1.47 ↑	1.63 ↑	1.20 ↑	1.42 ↑	1.38 ↑
TR_0_/CS_m_	0.93 ns	0.46 ↓	0.44 ↓	1.26 ↑	1.09 ns	0.93 ns
ET_0_/CS_m_	0.87 ns	0.31 ↓	0.30 ↓	1.39 ↑	0.91 ns	0.67 ↓
RE_0_/CS_m_	0.71 ↓	0.74 ↓	0.73 ↓	1.08 ns	0.82 ns	0.58 ↓

Note: 5 d, 10 d, 20 d, 30 d, 40 d, 50 d, and 60 d mean the ratio of flooding to control on days 0, 5, 10, 20, 30, 40, 50, and 60. ‘↑’, significantly increased. ‘↓’, significantly decreased. ‘ns’, not significant.

**Table 4:** The impact of flooding on physiological parameters in *Q. nattallii*.

Physiological Indexes	5 d	10 d	20 d	30 d	40 d	50 d	60 d
MDA	0.92 ns	1.35 ↑	1.52 ↑	2.06 ↑	2.47 ↑	1.97 ↑	1.99 ↑
H_2_O_2_	1.16 ↑	1.15 ↑	1.37 ↑	1.42 ↑	1.23 ↑	1.12 ↑	1.12 ↑
Soluble sugar	1.11 ns	1.11 ns	1.06 ns	1.38 ↑	1.29 ↑	1.36 ↑	1.11 ns
SOD	1.18 ↑	1.33 ↑	1.18 ↑	1.26 ↑	1.20 ↑	1.26 ↑	1.35 ↑
POD	1.08 ↑	1.20 ↑	1.35 ↑	1.28 ↑	1.42 ↑	1.45 ↑	1.24 ↑
ADH	1.12 ↑	1.13 ↑	1.39 ↑	1.12 ↑	1.20 ↑	1.48 ↑	1.32 ↑
IAA	0.90 ↓	0.68 ↓	0.75 ↓	0.64 ↓	0.68 ↓	0.64 ↓	0.58 ↓
GA_3_	0.61 ↓	0.62 ↓	0.90 ↓	0.83 ns	0.87 ↓	0.86 ↓	0.93 ns
ABA	1.30 ↑	1.32 ↑	2.34 ↑	1.29 ns	1.39 ↑	1.91 ↑	1.97 ↑
JA	0.84 ↓	0.61 ↓	0.66 ↓	1.27 ↑	1.09 ns	1.40 ↑	1.35 ↑

Note: 5 d, 10 d, 20 d, 30 d, 40 d, 50 d, and 60 d mean the ratio of flooding to control on days 0, 5, 10, 20, 30, 40, 50, and 60. ‘↑’, significantly increased. ‘↓’, significantly decreased. ‘ns’, not significant.

**Table 5:** The impact of flooding on physiological parameters in *Q. palustris*.

Physiological Indexes	5 d	10 d	20 d	30 d	40 d	50 d	60 d
MDA	1.38 ↑	1.24 ↑	1.20 ns	1.14 ↑	1.29 ns	1.29 ns	1.58 ↑
H_2_O_2_	1.01 ns	1.07 ↑	1.17 ↑	1.11 ↑	1.05 ns	1.10 ↑	1.25 ↑
Soluble sugar	1.17 ↑	1.04 ns	1.05 ns	1.17 ↑	1.32 ↑	0.98 ns	1.05 ns
SOD	1.18 ↑	1.20 ↑	1.04 ns	1.01 ns	1.03 ns	1.03 ns	1.15 ↑
POD	1.04 ns	1.00 ns	1.05 ns	1.26 ↑	1.11 ↑	1.14 ↑	1.12 ↑
ADH	1.05 ns	1.06 ns	1.18 ↑	1.18 ↑	1.17 ns	1.09 ↑	1.46 ↑
IAA	0.67 ↓	0.95 ns	0.76 ↓	0.62 ↓	0.46 ↓	0.48 ↓	0.48 ↓
GA_3_	1.03 ns	0.71 ↓	0.65 ↓	0.62 ↓	0.70 ↓	0.65 ↓	0.74 ns
ABA	1.35 ↑	1.97 ↑	1.53 ↑	2.29 ↑	1.60 ↑	1.40 ↑	1.40 ↑
JA	0.95 ns	1.03 ns	1.01 ns	1.14 ns	1.12 ↑	1.41 ↑	1.40 ↑

Note: 5 d, 10 d, 20 d, 30 d, 40 d, 50 d, and 60 d mean the ratio of flooding to control on days 0, 5, 10, 20, 30, 40, 50, and 60. ‘↑’, significantly increased. ‘↓’, significantly decreased. ‘ns’, not significant.

## Data Availability

All data used in this study are available within the paper and its Supplementary Materials published online.

## Supplementary Materials

The following supporting information can be downloaded at https://www.mdpi.com/article/10.3390/plants13121658/s1. Supplemental Table S1: Pearson correlation coefficients of photosynthetic and physiological parameters in *Q. nuttallii*. Supplemental Table S2: Pearson correlation coefficients of photosynthetic and physiological parameters in *Q. palustris*. Supplemental Table S3: Variable loading scores of physiological parameters and the proportion of variation in each principal component in *Q. nutallii* in response to flooding stress in different periods. Supplemental Table S4: Variable loading scores of physiological parameters and the proportion of variation in each principal component in *Q. palustris* in response to flooding stress in different periods.

## Abbreviations

L*, lightness. a*, red–green value. b*, yellow–blue value. a/b, the ratio of red–green value to yellow–blue value. C*, chroma value. ΔE, the overall color difference. P_n_, maximum net photosynthetic rate. T_r_, transpiration rate. C_i_, intercellular CO_2_ concentration. G_s_, stomatal conductance. WUE_i,_ instantaneous water use efficiency. F_v_/F_m_, the maximum efficiency of PSII. ABS/CS_m_, the absorption flux per unit area. DI_0_/CS_m_, the energy per unit area used for heat dissipation. TR_0_/CS_m_, the trapped energy flux per unit area. ET_0_/CS_m_, the energy per unit area used for electron transport. RE_0_/CS_m_, the number of reaction centers per unit area. MDA, malondialdehyde. H_2_O_2_, hydrogen peroxide. Ss, soluble sugar. SOD, superoxide dismutase. POD, peroxidase. ADH, alcohol dehydrogenase. IAA, included indole-3-acetic acid. GA_3_, gibberellic acid. ABA, abscisic acid. JA, jasmonic acid.
